# A Study on Dysmenorrhea and Its Self-Care Behaviors Among Women of Reproductive Age in Tiruvallur District, Tamil Nadu

**DOI:** 10.7759/cureus.71181

**Published:** 2024-10-10

**Authors:** Charumathi B, Priya P, Rowland Marlina

**Affiliations:** 1 Community Medicine, Saveetha Medical College and Hospital, Saveetha Institute of Medical and Technical Sciences (SIMATS), Saveetha University, Chennai, IND

**Keywords:** cross-sectional study, dysmenorrhea, prevalence, reproductive age group, self-care practices

## Abstract

Introduction: Menstrual cramps, which can be categorized as primary or secondary depending on the etiology, are a painful feature of dysmenorrhea. Researchers from throughout the country found that self-care practices for dysmenorrhea included reducing physical activity, adjusting diet, medications, or treatments, adopting alternative therapies, recording symptom clusters of discomforts, and expressing feelings.

Methodology: There were 246 women in the reproductive age range who took part in this study. Thiruvallur district's Thirumazhisai town panchayat served as the location of a community-based cross-sectional study. Parameters such as menstrual history, food history, self-care techniques during dysmenorrhea, and sociodemographic information were evaluated using a pre-tested semi-structured questionnaire.

Results: The prevalence of dysmenorrhea was found to be 71.54%. Among 47.15% of the participants, 75.8% who were married were found to have dysmenorrhea, whereas 67.7% of the 52.54% participants who were unmarried had dysmenorrhea. The association between age, smoking habits, exercise, and the presence of dysmenorrhea was found to be statistically significant.

Conclusion: Women, especially in India, have certain taboos and misconceptions associated with menstruation which can hurt their hygiene and self-care practices. This study highlights the need for menstrual hygiene awareness among women and proper self-care methods to be followed.

## Introduction

Dysmenorrhea, characterized by painful menstrual cramps, is classified into two types based on its etiology: primary dysmenorrhea, which has no well-established cause, and secondary dysmenorrhea, characterized by underlying pelvic pathology. The pathogenesis of dysmenorrhea is often attributed to elevated levels of PGF2a and PGF2, leading to increased uterine tone and contractions [1]. Women experience approximately 400 menstrual cycles throughout their reproductive years, equating to roughly 67 months or 5.5 years of menstruation [2]. The prevalence of dysmenorrhea varies considerably across different populations, with studies reporting rates ranging from 28% to 71.7% [3,4]. This wide range underscores the need for region-specific investigations to understand the condition's impact and prevalence better.

Self-care, defined as activities that individuals initiate and perform to maintain their health and well-being [5], plays a crucial role in managing dysmenorrhea. Common self-care behaviors reported in various studies include reducing physical activity, modifying diet, using medications or remedies, applying complementary therapies, monitoring symptom clusters, and expressing emotions [6]. In countries like India, where strong cultural taboos surrounding menstruation persist, these self-care practices may be significantly influenced by societal norms and beliefs [7,8]. The impact of dysmenorrhea extends beyond physical discomfort, often affecting daily activities, work productivity, and overall quality of life. This gap in care highlights the importance of understanding not only the prevalence of dysmenorrhea but also the self-care strategies employed by women to manage their symptoms.

Given the cultural context and potential barriers to seeking medical care in India, investigating the prevalence of dysmenorrhea and associated self-care behaviors is particularly relevant. This study aims to determine the prevalence of dysmenorrhea among women of reproductive age in Thirumazhisai, Thiruvallur district, identify factors associated with dysmenorrhea, and assess the self-care practices among women experiencing this condition. By exploring these aspects, we hope to contribute valuable insights that can inform public health strategies and improve menstrual health education in the region.

## Materials and methods

This community-based cross-sectional analytical study was conducted in Thirumazhisai, a town panchayat in the Thiruvallur district of Tamil Nadu, India, from July 2023 to September 2023. The study population comprised women of reproductive age (18 to 49 years) residing in the area. The sample size was determined using the formula N = 4PQ / L2, where P represents the prevalence of dysmenorrhea (45%) from a previous study [8], Q equals 100-P (55%), and L is 10% of P. This calculation yielded a required sample size of 246 participants. Participants were selected using simple random sampling from a comprehensive list of eligible women obtained through periodic family surveys conducted in urban field practice areas. Ethical approval was granted by the Saveetha Medical College and Hospital (SMCH) Institutional Ethics Committee [250/06/2023/IEC/SMCH]. Written informed consent was obtained from all participants before study enrollment.

Data collection was conducted using a pre-tested, semi-structured questionnaire that underwent pilot testing to ensure both validity and reliability. Trained female interviewers administered the questionnaire in the local language to promote accurate comprehension and responses from participants. The questionnaire was organized into four primary sections. The first section focused on sociodemographic characteristics, collecting data on variables such as age, marital status, educational attainment, occupational status, monthly income, socioeconomic status (using a specific scale such as the Kuppuswamy scale), religious affiliation, personal habits (such as smoking and alcohol consumption), and anthropometric measurements (including height and weight for BMI calculation). The second section addressed menstrual history, asking participants about their age at menarche, menstrual cycle duration and regularity, and menstrual hygiene practices, such as the number of sanitary pads used per day. It also included information on dysmenorrhea, covering aspects such as the presence, duration, and onset of pain in relation to the menstrual cycle. The severity of dysmenorrhea was assessed using a validated multi-dimensional scoring system. The third section gathered information on dietary habits, with a particular focus on daily consumption patterns of caffeine and tea. Participants were asked to recall their average daily intake of these beverages over the past month. The final section explored dysmenorrhea self-care strategies, divided into pharmacological and non-pharmacological approaches.

To ensure data quality, 10% of the questionnaires were randomly selected for re-administration by a supervisor. Any discrepancies were resolved through discussion or by revisiting the participant if necessary. Anthropometric measurements were taken using standardized equipment and procedures. Height was measured to the nearest 0.1 cm using a stadiometer, and weight was measured to the nearest 0.1 kg using a calibrated digital scale. BMI was calculated as weight in kilograms divided by height in meters squared. Statistical analysis was performed using IBM SPSS Statistics for Windows, Version 29 (Released 2023; IBM Corp., Armonk, New York, United States). Descriptive statistics were computed for all variables, with continuous data presented as means and standard deviations, and categorical data as frequencies and percentages. Associations between study variables and dysmenorrhea were assessed using chi-square tests for categorical variables and independent t-tests for continuous variables. Multiple logistic regression analysis was conducted to identify independent risk factors for dysmenorrhea. For all statistical tests, the appropriate test statistic (chi-square value, t-value, or F-value) was calculated alongside the p-value. A p-value < 0.05 was considered statistically significant.

## Results

The study included 246 women of reproductive age. Table 1 presents the sociodemographic characteristics of the participants. The mean age was 27.15 years, with a majority (79.66%, n=196) under 35 years old. Hinduism was the predominant religion (71.13%, n=175), followed by Christianity (14.63%, n=36) and Islam (14.23%, n=35). Educational attainment varied, with 26.42% (n=65) having completed graduate-level education and 23.57% (n=58) holding diploma or intermediate qualifications. Regarding employment status, 46.74% (n=115) were employed, 36.58% (n=90) were unemployed, and 16.66% (n=41) identified as housewives. Monthly income distribution revealed that 46.34% (n=114) earned less than 20,000 INR, while only 9.27% (n=23) earned 80,000 INR or more.

**Table 1:** Sociodemographic details of the study participants

Variables	Frequency (N=246)	Percentage (%)
Age		
<35 years	196	79.66
35 years and above	50	20.33
Religion		
Hindu	175	71.13
Muslim	35	14.23
Christian	36	14.63
Marital status		
Married	116	47.15
Unmarried	130	52.84
Education		
Post-graduate	28	11.38
Graduate	65	26.42
Diploma/ Intermediate	58	23.57
High school	44	17.88
Middle school	24	9.75
Primary school	14	5.69
Illiterate	13	5.28
Occupation		
Housewife	41	16.66
Employed	115	46.74
Unemployed	90	36.58
Monthly Income (INR)		
80,000	23	9.27
50,000-80,000	43	17.33
20,000-50,000	66	26.61
<20,000	114	46.34

Table 2 illustrates the lifestyle and behavioral characteristics of the participants. Smoking was reported by 10.16% (n=25) of the sample, with the majority of smokers (10.56%, n=26) having smoked for less than five years. Alcohol consumption was observed in 19.51% (n=48) of participants. Regarding physical activity, 51.62% (n=127) reported exercising five times per week, while 10.16% (n=25) did not exercise at all. Sleep patterns showed that 54.47% of participants got less than seven hours of sleep per night. Coffee consumption varied, with 54.47% consuming fewer than three cups daily, 34.55% not drinking coffee at all, and 10.97% consuming more than three cups per day. Tea consumption patterns revealed that 43.08% drank fewer than three cups per day, while 23.57% consumed more than three cups daily.

**Table 2:** Distribution of lifestyle and behavioral history among study participants

Variables	Frequency (N=246)	Percentage (%)
Do you smoke?		
Yes	25	10.16
No	221	89.83
If yes, mention the duration of smoking (in years)		
>20 years	1	0.4
10-20 years	1	0.4
5-10 years	4	1.62
<5 years	26	10.56
Not applicable	214	86.99
If yes, how many cigarettes a day?		
>20	1	0.4
Oct-20	1	0.4
05-Oct	1	0.4
<5	28	11.38
Not applicable	215	87.39
Do you consume alcohol?		
Yes	48	19.51
No	198	80.48
If yes, how frequently?		
Daily	0	0
Weekly once	2	0.81
Monthly	7	2.84
Quarterly	2	0.81
6 monthly	9	3.65
Only on festive or any events	38	15.44
Not applicable	188	76.42
How frequently do you exercise/walk/ jog/cycling, etc. per week?		
Not at all	25	10.16
5 times/per week	127	51.62
<5 times per week	94	38.21

**Figure 1:**
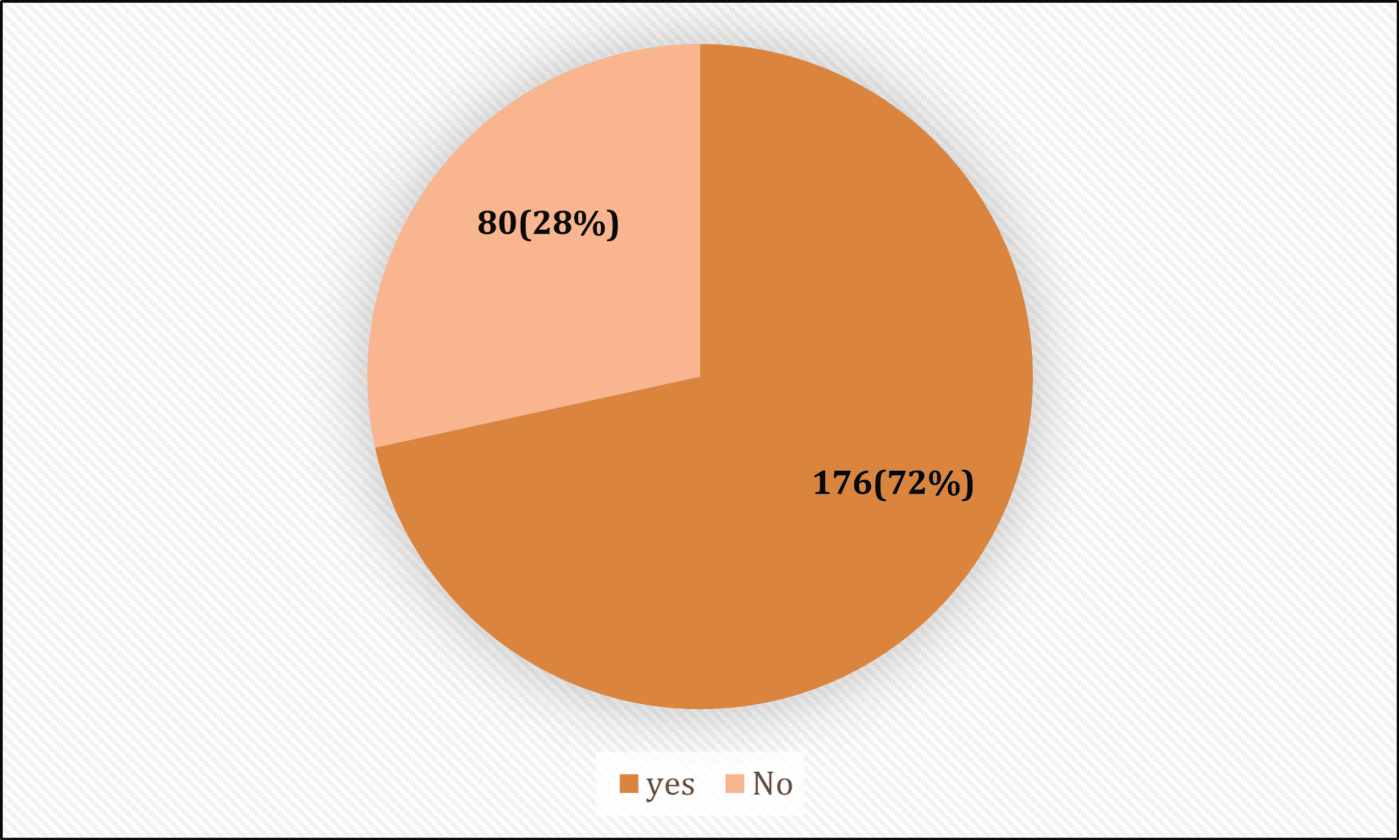
Prevalence of dysmenorrhea

**Figure 2:**
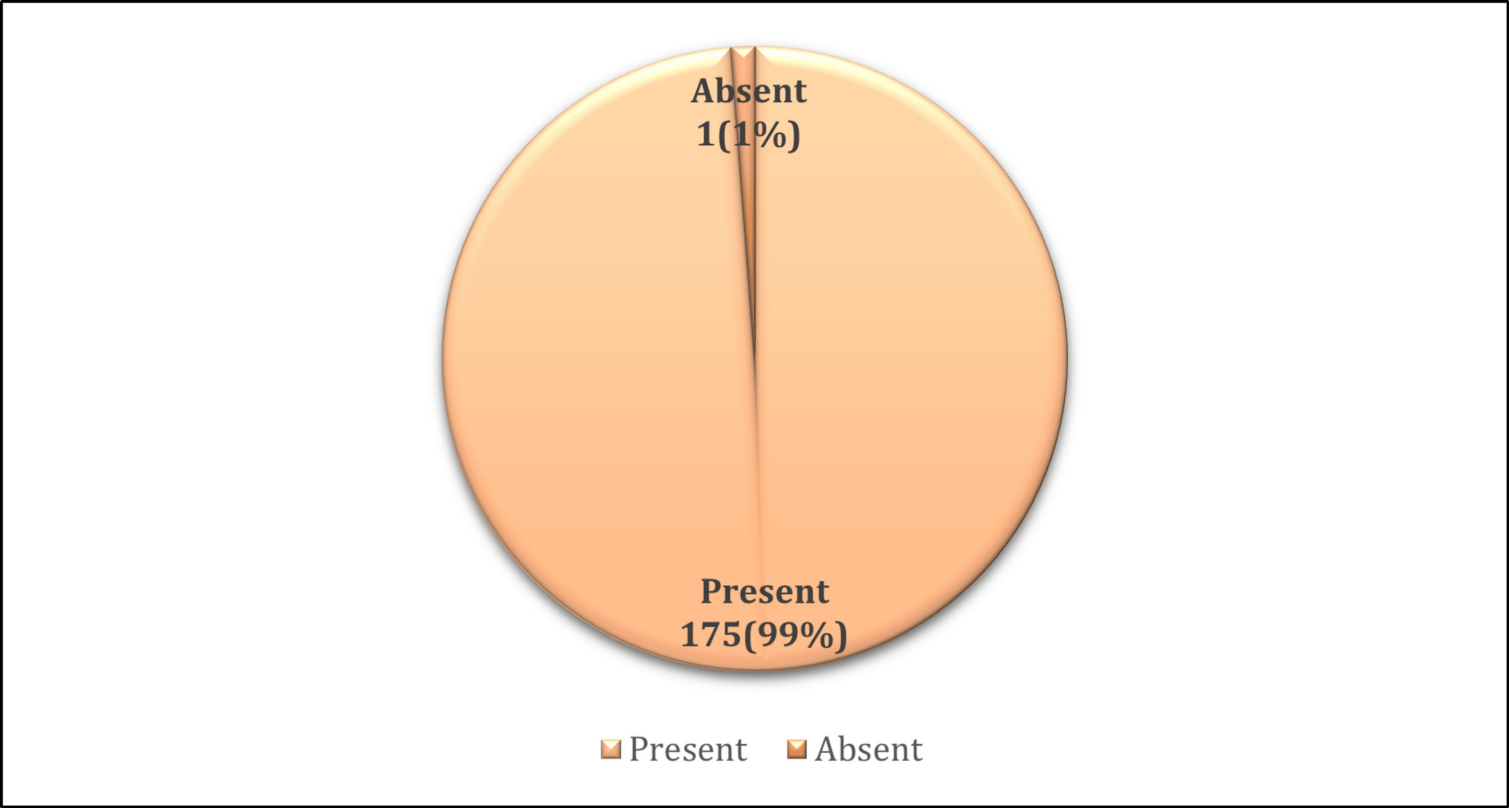
Associated symptoms with dysmenorrhea

The prevalence of dysmenorrhea among the study participants was 71.54% (n=176) (Figure 1). Table 3 presents the menstrual history of those experiencing dysmenorrhea. The majority reported cycles lasting 3-5 days, with 70.3% experiencing regular cycles. Notably, 48.37% of women with dysmenorrhea reported being moderately affected in their daily activities. The onset of dysmenorrhea varied among participants: 30% (n=53) had experienced it since menarche, 25% (n=44) within a year of menarche, and 20% (n=35) after a year of menarche. Figure 2 illustrates the associated symptoms of dysmenorrhea, indicating a wide range of experiences among affected individuals.

**Table 3:** Menstrual history of the study participants (n=176)

Variables	Frequency (N=176)	Percentage (%)
How long have you been experiencing dysmenorrhea? (in years)		
With menarche	53	30
Within a year of attaining menarche	44	25
After a year of attaining menarche	35	20
Three years ago	26	15
One year ago	18	10

Table 4 summarizes the self-care strategies and medical consultation patterns among women with dysmenorrhea. Regarding medical advice-seeking behavior, 28.97% (n=51) had never sought medical advice, while 60.79% (n=107) had done so occasionally. Self-medication was common, with 38.06% (n=67) sometimes using over-the-counter medications for symptom relief. However, only 18.18% (n=32) reported making changes to their diet or lifestyle during dysmenorrhea episodes. In terms of medical consultation, 36.3% visited private clinics, with gynecologists being the most frequently consulted specialists (39.7%). Notably, the vast majority (92.04%) did not consult traditional medicine practitioners.

**Table 4:** Self-care pharmacological and non-pharmacological strategies

Variables	Frequency (N=176)	Percentage (%)
Have you sought medical advice for dysmenorrhea?		
Never	51	28.97
Sometimes	107	60.79
Always	10	5.68
Once before	8	4.54
Have you self-medicated to relieve symptoms of dysmenorrhoea?		
Not at all	26	14.77
Sometimes	67	38.06
Always	31	17.61
Once before	52	29.54
Did you at any time change any of your diet or lifestyle during the period of dysmenorrhoea?		
Yes	32	18.18
No	144	81.81

Table 5 presents the associations between sociodemographic variables and dysmenorrhea. Age was significantly associated with dysmenorrhea (χ² = 13.14, p < 0.001), with younger women (<35 years) more likely to experience the condition. Marital status showed a trend towards significance (χ² = 2.07, p = 0.15), with 75.8% of married women reporting dysmenorrhea compared to 67.6% of unmarried women. Occupation approached statistical significance (χ² = 5.63, p = 0.06), suggesting that housewives (85.3%) might be more affected by dysmenorrhea compared to employed (71.3%) or unemployed (65.5%) women.

**Table 5:** Association between sociodemographic variables and dysmenorrhea (n= 246) χ²: Chi-square test statistic *p-value < 0.05 is considered statistically significant.

Variables	Dysmenorrhea present (N=176)	Dysmenorrhea absent (N=70)	Test statistic	p-value
Age			χ² = 13.14	0.00*
<35 years	142(72.4%)	54(27.5%)		
35 years and above	34(68%)	16(32%)		
Religion			χ² = 1.93	0.38
Hindu	121(69.1%)	54(30.8%)		
Muslim	26(74.2%)	9(25.7%)		
Christian	29(80.5%)	7(19.4%)		
Marital status			χ² = 2.07	0.15
Married	88(75.8%)	28(24.1%)		
Unmarried	88(67.6%)	42(32.3%)		
Education			χ² = 3.37	0.76
Post-graduate course	18(64.2%)	10(35.7%)		
Graduate	50(76.9%)	15(23%)		
Diploma/ Intermediate	42(72.4%)	16(27.5%)		
High school	30(68.1%)	14(31.8%)		
Middle school	20(66.6%)	10(33.3%)		
Primary school	16(76.1%)	5(23.8%)		
Occupation			χ² = 5.63	0.06
Housewife	35(85.3%)	6(14.6%)		
Employed	82(71.3%)	33(28.6%)		
Unemployed	59(65.5%)	31(34.4%)		

Table 6 illustrates the relationships between personal history factors and dysmenorrhea. Smoking status was significantly associated with dysmenorrhea (χ² = 4.18, p = 0.04), with 88% of smokers reporting the condition compared to 69.2% of non-smokers. Exercise frequency also showed a significant association (χ² = 9.21, p = 0.01), indicating that regular exercise (five times a week) was associated with a lower prevalence of dysmenorrhea. Tea consumption approached statistical significance (χ² = 5.99, p = 0.05), suggesting a potential relationship with dysmenorrhea

**Table 6:** Association between personal history and dysmenorrhea (n= 246) χ²: Chi-square test statistic; t: t-test statistic *p-value < 0.05 is considered statistically significant

Variables	Dysmenorrhea present (N=176)	Dysmenorrhea absent (N=70)	Test statistic	p-value
Do you smoke?			χ² = 4.18	0.04*
Yes	22(88%)	3(12%)		
No	153(69.2%)	68(30.7%)		
Do you consume alcohol?			χ² = 1.57	0.21
Yes	38(79.1%)	10(20.8%)		
No	139(70.2%)	59(29.7%)		
Duration of sleep (in hours)			t = 0.61	0.54
<7 hours	98(73.1%)	36(26.8%)		
>7 hours	78(69.6%)	34(30.3%)		
How many cups of coffee do you take per day?			χ² = 3.66	0.16
Not at all	55(64.7%)	30(35.2%)		
<3	99(73.8%)	35(26.1%)		
>3	22(81.4%)	5(18.5%)		
How many cups of tea do you take per day?			χ² = 5.99	0.05
Not at all	51(62.1%)	31(37.8%)		
<3	83(78.3%)	23(21.6%)		
>3	42(72.4%)	16(27.5%)		
How frequently do you exercise/walk/ jog/cycling, etc. per week?			χ² = 9.21	0.01*
Not at all	12(48%)	13(52%)		
5 times/per week	96(75.5%)	31(24.4%)		
<5 times per week	68(72.3%)	26(20.4%)		

Overall, this study found a high prevalence of dysmenorrhea among women of reproductive age, with several sociodemographic and lifestyle factors significantly associated with its occurrence. These findings highlight the need for targeted interventions and education programs to address this common condition

## Discussion

This cross-sectional study, conducted among 246 women of reproductive age in the Thiruvallur district of Tamil Nadu, India, revealed a high prevalence of dysmenorrhea (71.54%). This finding aligns with previous research, such as the study by Al-Qazaz et al., which reported a prevalence of 93.6% [9]. The variation in prevalence may be attributed to differences in study populations and methodologies.

Dysmenorrhea significantly impacts the quality of life for many women and is often linked to a chronic condition that causes persistent disruption in daily activities [10]. Our study corroborates this, with 76.7% of women reporting moderate to severe effects on their daily functions, a statistically significant finding (p<0.01). The majority of women in our study experienced dysmenorrhea from the onset of menarche, suggesting a predominance of primary dysmenorrhea. This aligns with the understanding that primary dysmenorrhea is related to normal ovulatory cycles without pelvic pathology [11].

Regarding self-care behaviors, our study found that 66.3% of women sought medical advice for dysmenorrhea, primarily from general practitioners or gynecologists. This rate is considerably higher than the 11.33% reported by Rani et al. in Chandigarh, India [12]. The disparity may reflect regional differences in healthcare access, health literacy, or cultural attitudes toward menstrual health. Balamurugan et al. emphasized the importance of improving female literacy and health education regarding menstrual hygiene, particularly in rural populations [13]. Non-pharmacological management strategies, such as consuming warm drinks, bed rest, and reduced physical activity, were commonly reported in our study. However, these methods may not always be practical, especially during work hours or travel. In such cases, the use of NSAIDs or other medications for temporary pain relief becomes crucial [14]. Interestingly, 18.8% of women in our study reported modifying their diet or lifestyle during dysmenorrhea, a finding that warrants further investigation into the potential benefits of dietary interventions.

Our study revealed significant associations between dysmenorrhea and factors such as age, smoking status, and exercise frequency. Younger women (<35 years) were more likely to experience dysmenorrhea, consistent with previous findings [15]. The association between smoking and increased prevalence of dysmenorrhea aligns with research by Omidvar et al., who reported that unhealthy lifestyle factors, including excessive caffeine intake, increase the risk of menstrual disorders [16]. Regular exercise was associated with a lower prevalence of dysmenorrhea in our study. This finding supports growing evidence that physical activity may help alleviate menstrual pain, possibly through endorphin release and improved pelvic blood flow [17]. The implications of these findings are significant for developing targeted interventions and education programs to address this common condition.

Several limitations should be acknowledged when interpreting the findings of this study. First, the cross-sectional design limits the ability to establish causal relationships between the identified factors and dysmenorrhea. Second, the reliance on self-reported data introduces the possibility of recall bias, which may affect the accuracy of the responses. Future research could address this issue by incorporating more objective measures. Third, the study's focus on a specific geographical area, Thirumazhisai in the Thiruvallur district, may restrict the generalizability of the findings to populations with different socio-cultural contexts or healthcare systems. Additionally, while the study examined various factors associated with dysmenorrhea, it did not distinguish between primary and secondary dysmenorrhea, which could have distinct risk factors and management strategies. Moreover, the sample size, though sufficient for the study's primary objectives, may have been underpowered to detect associations with less common risk factors. Despite these limitations, this study offers valuable baseline data on dysmenorrhea in the population studied and underscores important areas for future research and intervention.

## Conclusions

This study highlights the significant prevalence and impact of dysmenorrhea among women of reproductive age in Thiruvallur district, Tamil Nadu. The findings reveal that over 70% of women experience dysmenorrhea, with a considerable proportion reporting moderate to severe effects on daily activities. Younger age, smoking, and lack of regular exercise were identified as key factors associated with increased prevalence of dysmenorrhea. The study also uncovered important insights into self-care behaviors and healthcare-seeking patterns, with a majority of women using non-pharmacological methods for pain relief and a significant proportion seeking medical advice. These results underscore the need for comprehensive menstrual health education programs, particularly targeting younger women and addressing modifiable risk factors such as smoking and physical inactivity. Healthcare providers should be aware of the high prevalence of dysmenorrhea and be prepared to offer evidence-based management strategies. Future research should focus on developing and evaluating targeted interventions to reduce the burden of dysmenorrhea and improve overall menstrual health. Ultimately, addressing dysmenorrhea effectively could significantly enhance the quality of life and productivity of women in this region and beyond.
